# The Hand Eczema Trial (HET): design of a randomised clinical trial of the effect of classification and individual counselling versus no intervention among health-care workers with hand eczema

**DOI:** 10.1186/1471-5945-10-8

**Published:** 2010-08-31

**Authors:** Kristina Sophie Ibler, Tove Agner, Jane Lindschou Hansen, Christian Gluud

**Affiliations:** 1 Department of Dermatology, Roskilde Hospital, Roskilde, Denmark; 2 Department of Dermatology, Bispebjerg Hospital, Copenhagen, Denmark; 3 MSc Demography and Health, Copenhagen Trial Unit, Centre for Clinical Intervention Research, Rigshospitalet, Copenhagen, Denmark; 4 Copenhagen Trial Unit, Centre for Clinical Intervention Research, Rigshospitalet, Copenhagen, Denmark

## Abstract

**Background:**

Hand eczema is the most frequently recognized occupational disease in Denmark with an incidence of approximately 0.32 per 1000 person-years. Consequences of hand eczema include chronic severe eczema, prolonged sick leave, unemployment, and impaired quality of life. New preventive strategies are needed to reduce occupational hand eczema.

**Methods/Design:**

We describe the design of a randomised clinical trial to investigate the effects of classification of hand eczema plus individual counselling versus no intervention. The trial includes health-care workers with hand eczema identified from a self-administered questionnaire delivered to 3181 health-care workers in three Danish hospitals. The questionnaire identifies the prevalence of hand eczema, knowledge of skin-protection, and exposures that can lead to hand eczema. At entry, all participants are assessed regarding: disease severity (Hand Eczema Severity Index); self-evaluated disease severity; number of eruptions; quality of life; skin protective behaviour, and knowledge of skin protection. The patients are centrally randomised to intervention versus no intervention 1:1 stratified for hospital, profession, and severity score. The experimental group undergoes patch and prick testing; classification of the hand eczema; demonstration of hand washing and appliance of emollients; individual counselling, and a skin-care programme. The control group receives no intervention. All participants are reassessed after six months. The primary outcome is observer-blinded assessment of disease severity and the secondary outcomes are unblinded assessments of disease severity; number of eruptions; knowledge of skin protection; skin-protective behaviour, and quality of life.

**Trial registration:**

The trial is registered in ClinicalTrials.Gov, NCT01012453.

## Background

Hand eczema (HE) is a long-lasting disease with a point prevalence of 9.7% in the background population [1] and an incidence reported to be 5.5 to 8.8 per 1000 person-years [2,3]. Occupational hand eczema (OHE) is the most frequently recognized occupational disease in Denmark with an incidence of approximately 0.32 per 1000 person-years [4]. Other studies have revealed that the annual incidence of new reports of occupational skin diseases is 0.7 to 0.8 per 1,000 employees [5,3] and the number of unreported occupational skin conditions are many times greater. Despite governmental attempts to reduce exposures to harmful occupational allergens, the number of new OHE patients has remained almost unchanged during the past decade [4]. The prevalence is highest in females aged 20-30 years and there is an increased risk in occupations with high exposure to wet work, skin irritants, and contact allergens [1,2]. Complications and consequences of occupational hand eczema include chronic severe eczema, prolonged sick leave, unemployment, and impaired quality of life [6-10].

In Denmark, 21% of the recognized occupational skin diseases are represented by health-care workers [11]. Nurses, assistant nurses, and nursing aids are particularly at high risk, about a third reporting hand eczema [12].

Among factors that can lead to OHE are wet work with frequent hand washing, use of protective gloves, and local disinfectants [13-15]. There are no data available on the quantitative exposure to wet work in the different specialties and professions in a hospital. Better methods to assess the exposure to wet work are needed, and information on allergens and irritants related to development of hand eczema is lacking [16].

### Clinical data

Preventive measures and skin-care programmes have shown a significant positive effect in the prevention of HE among health-care workers [17-20], and a recent study on Danish health-care workers shows that preventive efforts are necessary in hospitals [12]. Skin-care programmes have also been effective in studies of other occupations such as hairdressers [21,22], gut cleaners [23], and cheese dairy industry workers [24]. Several of the mentioned trials were conducted as cluster randomised trials [17-21,23,24] and assessed primary prevention [17-20,23,24].

Secondary prevention of HE in individual geriatric nurses was examined in Germany in 2004 [25]. The participants were initially referred to the authorities (Berufsgenossenschaft fur Gesundheitsdienst und Wohlfahrtspflege, BGW) by their local dermatologist who suspected occupational skin disease. All participants were interviewed prior to trial initiation. The intervention was complex and comprised four visits in six months including one-to-one consultation by a dermatologist, three educational seminars with hands-on training in the correct use of skin protection and dermatologic treatment by en educationalist focusing on attitudes toward illnesses and motivation to remain at work. At each visit Δ transepidermal water loss (Δ TEWL) was measured. The intervention resulted in improvement in objective dermatologic findings and skin physiologic data (TEWL).

In Germany there is a special course of action for health-care workers with occupational skin disease [26]. Whenever a skin disease is reported to the BGW, the patient is immediately invited to attend a 2-day skin protection course that is organised in cooperation with dermatologists/allergists, specialists in occupational medicine, hygiene specialists, and BGW staff members. The educational part of the skin protection courses is complemented by a medical part obtained by a dermatologist in each patient. This comprises medical history including atopic dermatitis, further diagnostics, therapy, skin protection, and an assessment of whether the patient can remain in the job. The findings are sent to the BGW who decide how to handle the patient in the future. This can vary from initiation of a 3-weeks inpatient treatment programme (tertiary inpatient individual prevention programme) to initiation of an advisory/expert's opinion. A follow-up investigation based on telephone interviews on 206 of 253 health-care workers showed that the skin lesions had decreased significantly, skin care and skin protection had improved, while the frequency of reported hand washing was reduced. A significantly positive impact on quality of life was also observed [27].

With respect to secondary prevention of HE, valid randomised clinical trials are lacking. Treatments are often used without differentiation between HE subtypes, and only a few clinical studies have identified subtypes [28-33]. According to morphology and aetiology, HE can be divided in the following subtypes: allergic contact dermatitis (ACD), irritant contact dermatitis (ICD), atopic HE (AHE), vesicular HE, hyperkeratotic and discoid HE. Combinations of the subtypes exist of which ACD and ICD are the most common followed by AHE and ICD [33]. In order to establish an effective prevention programme it is necessary to understand the aetiology of HE. In a Danish study on patients with OHE, irritant contact dermatitis was found to occur more frequently than allergic contact dermatitis [34]. This was also found in a recent German study on geriatric nurses from nursing homes and home care facilities [35] and in a study on 1301 health-care workers from 1995 [36]. On the contrary, an English study on nurses with OHE found that allergic contact dermatitis was more common [37]. Previous studies indicate that the patient's knowledge of the disease (HE) is important for the prognosis of the disease [38,39].

The effect of a prevention programme consisting of a combination of classification of HE and individual, work-related counselling in skin protective behaviour, has not yet been investigated in Denmark. The HET trial is the first trial on secondary prevention that is individually randomised and stratified according to hospital (the three different hospitals involved), profession (physicians compared to nurses, nursing aids, and biotechnicians), and Hand Eczema Severity Index (HECSI) score.

## Methods and Design

### Trial participants

The trial participants are identified through a self-administered questionnaire comprising 3,181 health-care workers in three Danish hospitals in the same geographical region of the country. The questionnaire addresses the prevalence of HE, exposures and risk factors for development of HE in the different departments, duty hours, and professions. Furthermore, it addresses the knowledge of skin-protective behaviour among the health-care workers.

#### Inclusion criteria

- Participants who answered "yes" to the validated question "Have you had hand eczema within the past twelve months?"

- Informed written consent

#### Exclusion criteria

- Pregnancy

- Systemic use of immunosuppressive drugs

- Systemic use of retinoids

- Active psoriatic lesions on the hands

- Any serious medical condition which, in the opinion of the investigator, may interfere with the evaluation of the results

- Lack of informed written consent

### Design

**Figure 1:**
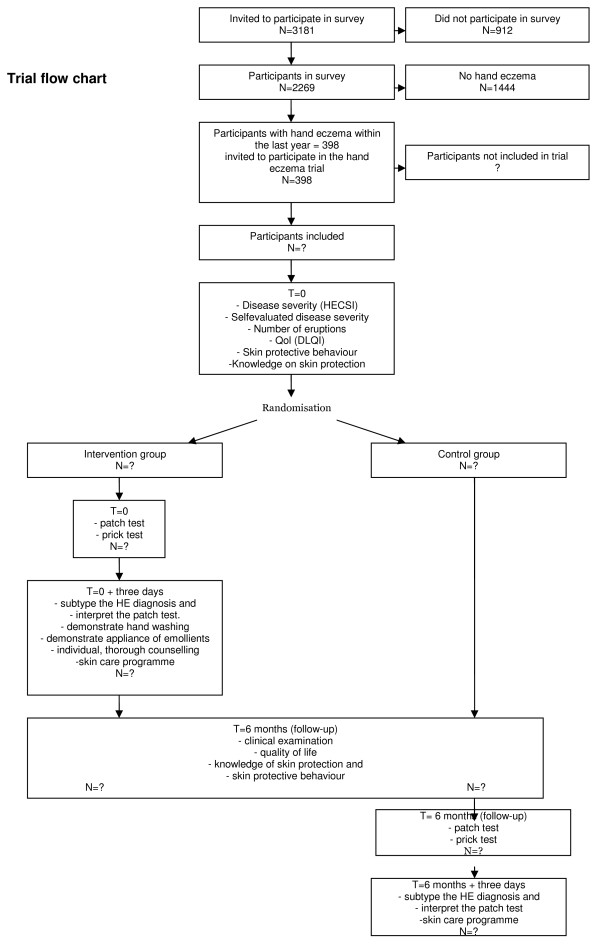
**Trial flow chart**.

HET is a randomised, observer-blinded parallel trial. All included participants are clinically examined at the beginning and at the six months follow-up in the trial. Half of the participants will be randomised to the experimental intervention, the other half to the control intervention consisting of no intervention. The participants in the experimental group will, after the first clinical examination, pass on directly to the intervention which includes an allergological examination (patch and prick testing). Three days later they will be examined by a physician who will interpret the patch test and give a thorough, individual guidance in skin protection and occupational safety. The clinical examination of all participants at six-months follow-up will examine the outcomes in the intervention and the control group (Figure 1).

### Randomisation

Randomisation will be individual and performed centrally at the Copenhagen Trial Unit (CTU) according to a computer generated allocation sequence with a block size unknown to the investigators. After a clinical examination, an investigator will contact the CTU by telephone and the CTU staff will randomise the participant to intervention or control group. The participants in the intervention group will then pass on directly to the intervention and they will be told not to share knowledge during the investigation. The percentage of participants allocated per intervention will be 50:50. Randomisation will be stratified according to three factors: hospital (the three hospitals involved), profession (physicians compared to nurses, nursing aids, and biotechnicians), and HECSI score at entry (HECSI <8 versus HECSI ≥8).

### Detailed description of the experimental intervention

A self-administered questionnaire was distributed to all nurses, physicians, nursing aids, and laboratory technicians in three Danish hospitals, 3,181 individuals in all. The questionnaire was based on a standardised questionnaire for work-related skin diseases and exposure - the Nordic Occupational Skin Questionnaire (NOSQ-2002) [38]. Wherever needed questions were modified to the hospital environment. The questionnaire was distributed electronically by email and those who did not respond were sent a paper version.

The questionnaire investigates the prevalence of HE among health-care workers and the risk factors/exposures (amount of hand washing, use of hand disinfectants, protective gloves, emollients, etc) related to different hospital wards (medical, surgical, in- and out patient wards), duty hours, working procedures, and professions. The participants with HE were asked about self-evaluated disease severity and knowledge of skin protection was evaluated through multiple choice questions. Questions on domestic exposures as well as allergic and atopic dispositions were also posed.

The questionnaire identified 398 individuals who answered "yes" to the question: "Have you had hand eczema within the past year?"[40]. These individuals will be invited by a personal letter to a clinical examination which will focus on *disease severity, self-evaluated disease severity, registration of eruptions through the past quarter*, and *quality of life*. Disease severity will be measured by the use of the HECSI score, which is a validated scoring system including scoring of erythema, infiltration, vesicles, fissures, scaling and oedema [41] as well as scoring of the size of the affected area. The HECSI score ranges from 0 (no HE) to 360 (maximum degree of HE). Self-evaluated disease severity will be reported by the participants by use of a validated photographic guide [42]. Number of eruptions through the past quarter will be reported as the number given by the participant. Quality of life will be registered by use of Dermatology Life Quality Index (DLQI) [43,44], a validated dermatology-specific questionnaire, which has previously proved useful for assessment of quality of life in patients with HE [10,45]. Skin protective behaviour will be studied by specific questions developed from a skin protection programme [46] and through information withdrawn from the questionnaire about daily handwashing, use of hand disinfectants, protective gloves, and emollients.

The person responsible for the clinical examination is a health-care person who has received training in the use of HECSI-score by a dermatologist. The time burden of the clinical examination is fifteen minutes.

After the clinical examination the participants are randomised to the intervention or the control group. The participants in the intervention group will pass on directly from the clinical examination to the intervention. They will be tested with a patch test *(allergens: nickel sulfate, wool alcohols, neomycin sulfate, potassium dichromate, caine mix, fragrance mix, colophony, paraben mix, negative control, balsam of peru, ethylenediamine dihydrochloride, cobalt dichloride, p-tert-butylphenol formaldehyde resin, epoxy resin, carba mix, Black Rubber mix, Cl+ Me- isothiazolinone, quaternium-15, mercaptobenzothiazole, p-phenylenediamine, formaldehyde, mercapto mix, thimerosal, thiuram mix, chlorhexidine digluconate 0.5%, primin 0.01% petrolatum, sesquiterpene lactone mix 0.1% petrolatum (pet), budesonide 0.01% pet, tixocortol pivalate 0.1% pet, hydroxyisohexyl-3-cyclohexene carboxaldehyde 5% pet, methyldibromo glutaronitrile 0.5% pet, fragrance mix II 14% pet) *and a prick test *(Alk-Abello soluprick standard series, chlorhexidine 0.5%, latex) *for relevant allergies that could explain the presence of HE. Patch and prick tests will be performed by a nurse. The time burden is fifteen minutes for the patch and prick testing.

Three days after application of the patch tests, a physician will interpret the patch test and subtype the HE diagnosis. The participants will demonstrate how they apply an emollient on their hands by use of a fluorescent lotion and UV-light (GlitterBug Potion) to detect areas where the lotion is not properly applied. Thereafter the emollient is washed off, and the investigator will register how the handwashing is performed by the participant. Correct instructions in handwashing/appliance of emollients will be given thereafter as well as individual, thorough counselling in occupational safety and skin protection based on a skin care programme [46] and individual information on protective behaviour. The time burden will be 20-30 minutes.

Any participant who presents in the trial with severe HE that needs medical treatment will be prescribed with moisturizers or local corticosteroids depending on severity. For further therapy, if needed, the participants will be referred to their general practitioner.

### Detailed description of the control intervention

The participant in the control group will undergo the questionnaire assessment as well as the initial clinical examination including HESCI score assessment.

Any participant who present in the trial with severe HE that needs medical treatment will be prescribed with moisturizers or local corticosteroids depending on severity. For further therapy, if needed, the participants will be referred to their general practitioner.

After the six-month follow-up individual counselling and patch and prick test will be offered to the control participants.

### Concomitant medications

#### Medication *not permitted *during the trial

Systemic immunosuppressive drugs (such as azathioprine, cyclosporine, or prednisolone).

Systemic retinoids.

#### Medication *permitted *during the trial

Local immunomodulators (such as corticosteroids, pimecrolimus, or tacrolimus).

All other medication that does not affect the immune system including rescue medication.

### Monitoring for participant compliance

Participant compliance will not be monitored during the intervention period.

### Follow-up at six months

All participants from the intervention group and the control group will have a clinical examination with registration of disease severity, self-evaluated disease severity, registration of eruptions through the past three months and quality of life by using the same instruments as mentioned at entry into the trial. Both groups will have a new questionnaire including multiple choice questions about knowledge of skin protection and questions on skin protective behaviour. The questions will be identical to those asked at entry to the trial.

After the clinical follow-up examination, the participants from the control group will be offered allergological patch test (European Standard Series; TRUE Test Panel 1 and 2 and chlorhexidine) and prick test (standard test, chlorhexidine and latex) applied by a nurse. Three days after appliance a physician will interpret patch test results, classify HE and give advise regarding relevant allergies, skin protection, and occupational safety.

### Blinding

The trial is observer blinded and involves three investigators. Investigator 1 (the outcome assessor) is responsible for the clinical examination at entry and follow-up and will, together with the statistician, be the only blinded persons in the trial. The randomisation and allocation will not be done until after the first clinical examination, and the participants will be told not to share information with investigator 1 at follow-up. Investigator 2 (a nurse) will be responsible for patch and prick tests at entry and follow-up. Investigator 3 (a physician) will be responsible for subtyping the HE, interpreting the patch test at entry and follow-up and counselling of the intervention group at entry, and the control group at follow-up. Investigator 3 will be administrating the interventions and assessing the secondary outcomes.

As randomisation is individual, there is a risk of sharing knowledge among the participants. To minimise this problem, the participants will be told not to share knowledge with colleagues during the trial.

### Intervention accountability

All necessary materials and tools will be handled as prescribed.

### Trial conduct

The trial will be conducted in compliance with the protocol approved by the Danish Data Protection Agency and the local ethics committee. No deviation from the protocol will be implemented without prior review and approval of these authorities.

### Trial objectives

The HET trial is based on the complex intervention of precise classification of HE, allergological investigation, and individual counselling compared with a control group receiving no intervention.

### Efficacy variables

The effects that are to be assessed in the trial are the following:

#### Primary outcome

Objective blinded assessment of disease severity (HE), measured as the difference in HECSI-score at follow-up minus the HECSI-score at time entry.

#### Secondary outcomes

Subjective assessment of disease severity (HE), measured by use of a photographic guide, at follow-up minus at entry.

Number of eruptions registered by the participant through the past three months of the trial at follow-up minus at entry.

Knowledge of skin protection measured as numbers of points achieved in a repeated multiple choice questionnaire on skin protection at follow-up minus at entry.

Skin protective behaviour measured as number of daily handwashing and use of hand disinfectants and emollients at follow-up minus at entry. Skin protective behaviour will also be measured as number of correct answers according to questions developed from a specific skin care programme at follow-up minus at entry.

Quality of life will be measured as number of points scored in the Dermatology Life Quality Index at follow-up minus at entry.

### Adverse events

Any undesirable event occurring to a participant during a clinical trial, whether or not related to the trial, is considered to be an adverse event.

Since no drugs are used in the trial, the only expected adverse events are unexpected reactions to patch and prick tests. These can be allergic and eczematous reactions with redness of the skin, vesicles, itching, and urticaria. However, these symptoms can be present as a normal, positive response to patch and prick testing. If the reactions are severe and long lasting with involvement of skin areas other than the tested areas, it will be considered as an adverse event.

Systemic reactions are rare and can be astma, pruritic eyes, nose or pharynx, generalised pruritus, sneezing, and generalised urticaria [47]. Anaphylactic reaction is extremely rare [48] and will be reported as a serious adverse event. Adverse and serious adverse events will be reported in compliance to the ethics committee requirements.

### Serious adverse events

Any serious adverse event will be registered. These include any experience that suggests a medically significant hazard including any event that: results in death; is life threatening; requires inpatient hospitalisation; results in persistent or significant disability/incapacity; is a congenital anomaly/birth defect.

### Recording of adverse events

At six months, all adverse events either observed by the investigator or reported by the participant will be recorded in the participants file by the investigator and evaluated. Following variables will be recorded: description of event, onset and end of event, severity, relation to intervention product, action taken and outcome.

### Type and duration of the follow up of participants after adverse events

Any adverse event occurring during the trial will be treated according to established standards and the participant will be followed until the event has disappeared or until the condition has been stabilised.

### Ethical considerations

The intentions with the experimental intervention are to improve behaviour and knowledge of skin protection among health-care workers in order to prevent HE in this population. No drugs will be used in the trial and the participants are not put on any unacceptable level of risk and there are no perceived harms connected to the trial other than disadvantages correlating to patch and prick testing described above. Patch and prick testing are established diagnostic procedures that are used in daily practise in dermatological clinics and departments.

All participants are offered a standard allergological investigation to detect relevant allergies that can have an impact on HE. However, participants in the intervention group will be allergologically tested at entry to the trial, and participants in the control group will be tested after follow-up. Thus the participants in the control group are allergologically diagnosed six months later than the intervention group, and this is considered to be the main ethical dilemma in the trial. During the intervention period the participants in the control group do not know if they are allergic, and therefore might not avoid the relevant allergens. This can have a negative impact on the eczema, but it will not differ from the conditions before onset of the trial. We do, however, consider that the collective advantages exceed the disadvantages for the population as a whole. By the end of the trial all participants have been treated equally. In Denmark there are at present no standard procedures or standard treatments when it comes to patients with HE, and the participants in the control group are not considered to be treated worse than 'usual standard care'. HE patients are followed by general practitioners, dermatologists in private practise, or dermatological departments. In general practise, allergological investigations are not done. In dermatological practise or departments allergological testing is usually done depending on the patient's history, the severity of the eczema, and treatment response. The treatment usually includes avoidance of wet work and irritants, use of emollients, and prescription of topical corticosteroids. Other treatments that can be used for HE include topical calcineurin inhibitors, oral steroids, azathioprine or cyclosporine, ultraviolet (UV) radiation, psoralene and UV-A radiation, alitretinoin, and retinoids.

### Participant information and informed consent

This HET protocol has been approved by the local Ethics Committee. All participants considered for this trial will be provided with written and oral information on the trial so that the participants can make an informed decision about their participation in this trial. The consent form will be signed by the participant and the investigator seeking the consent.

### Data collection

Data will be registered directly in standardised paper record forms at each visit by investigator 1, 2 and 3. All data from the paper files will be registered electronically in SPSS. This will be done manually by double data entry performed by the investigators:

At entry to the trial, all participants will have three coded case record forms to be used by investigator 1, 2 and 3. At follow-up, three new coded case records will be used.

### Participant withdrawal

The participants are free to withdraw his/her informed consent from the trial at any time without effecting future treatment. All participants who enter the trial will be accounted for in the report, whether or not they are included in the analysis. All reasons for exclusion from analysis will be documented. Participants who do not attend the follow-up visit will be identified and a letter will be sent containing a questionnaire on reasons for drop out and its relationship to treatment and outcome, on number of eruptions, subjective severity assessment, quality of life, knowledge of skinprotection, and skin protective behaviour. The questions will be identical to the questions asked at the clinical examination supplemented by questions on skin protective behaviour for the participants in the intervention group. If the participant does not respond to the letter, they will be contacted by telephone and asked to take part in a telephone interview by investigator 1. The telephone interview will include the same questions as in the letter. Information on objective severity assessment cannot be obtained since that demands a clinical examination. Subjective severity assessment can only be obtained by the use of the photographic guide that will be distributed in a letter.

### Sample size estimation

The clinical trial is planned to include a minimum of 262 participants. The sample size calculation is based on the mean HECSI score (primary outcome) after six months, which is expected to be 10 in the intervention group and 14 in the control group. Alpha error level is 5% and beta error level is 20%. With the standard deviation of 13 on the HESCI score, the sample size calculation is 131 each intervention group. http://www.dssresearch.com/toolkit/sscalc/size_a2.asp.

Since the prevalence of HE is approximately 10% in the health-care worker population, 3181 health-care workers were invited to participate in the questionnaire survey. This was the number of employed health-care workers (doctors, nurses, nursing aids and biotechnicians) in the three included hospitals. The results of the survey identified 398 health-care workers with HE during the past year. All 398 health-care workers are invited to join the trial. We do, however, not expect that all invited HCW will participate. The time span between the survey and the clinical trial is five months and there will be a natural drop out among the invited health-care workers.

### Statistical methods and significance

Statistical analysis will be performed in SPSS. Comparisons of quantitative exposures between different working conditions will be analysed using the Mann-Whitney test. Non-parametric statistics (Mann-Whitney test) will be used to compare independent groups. Changes between matched data over time (6 months) will be analysed through McNemar's test for dichotomy variables and test of marginal homogeneity will be used for ordinal data. The significance level will be a p value ≤ 0.05.

The number of participants included in the statistical analyses will be reported. We intend to conduct intention-to-treat analyses.

A trained statistician will guide in the statistical aspects of the trial, and all data analyses will be conducted with the statistician blinded for intervention groups.

### Accountability procedure for missing data/population for analysis

An analysis of dropouts will be made to describe the demographic data of the population. The analysis will be used to compare the drop outs with the participants and investigate whether the two groups differ in demographic conditions. If more than 5% of the data is missing, multiple imputation will be performed if data are not missing completely at random.

### Direct access to source data and documentation

The trial is not planned to be monitored by any other authority than the investigators. If a relevant authority, as the Danish Research Ethics Committee System or the Danish Data Protection Agency, plan to inspect the trial, all data and files will be available for inspection in accordance with the GCP guidelines.

### Data handling and record keeping

Data will be handled and recorded in case record forms and kept in records marked with investigator number, patient identification number, name of hospital, and time. After follow-up all the case report forms from each participant will be collected in individual files. Any change in the files or case report forms will be documented with date and signature of the investigator.

Data from the records will be registered electronically in SPSS for statistical analyses. This will be done manually. Records will be archived for at least five years after termination of the trial.

### Quality control and quality assurance

To ensure that the trial is conducted and reported in compliance with this protocol, the data will be monitored internally and externally. The investigators will monitor the data and check for systematic errors. All data will be registered in paper record forms and kept at an investigator file site only available for the investigators. The data will be registered electronically by the investigators using double data entry. A random 5% of the data will be monitored by the investigators. Data will be handled with confidentiality.

### Trial organisation

The trial takes place in three hospitals in Region Zealand who has provided the participants and localities for investigation. The Danish Working Environment Authority has been involved in the creation of the questionnaire.

The investigators are responsible for the protocol, conducting of the trial, and all other aspects involved.

### Finance and insurance

The trial is financed by the Danish Working Environment Authority and Region Zealand Health Scientific Research Foundation who cover all expenses related to the trial. The participants in the study are covered by their work insurance and the patient insurance (Patientforsikringen: http://www.patientforsikringen.dk)

## Discussion

The overall purpose of the HET trial is to develop new strategies for secondary prevention of HE in health-care workers. The trial focuses on HE among nurses and nursing aids who account for almost 25% of recognized OHE in Denmark.

The project will assess exposures that can lead to OHE in hospitals and relate them to different wards, duty hours, and professions. It also focuses on the knowledge of skin protection and skin protective behaviour in health-care workers, which may make it possible to improve preventive strategies.

The project will also investigate the aetiology of HE in health-care workers. Aetiology and assessment of exposures in health-care workers are important factors for focused prevention of HE in the future. This combined with individual, focused counselling could make a basis for a new strategy in prevention of HE in health-care workers.

We have designed the HET trial in order to reduce the risks of systematic errors ('bias'), random errors ('play of chance'), and design errors to a minimum [49-51]. The risks of bias have been sought reduced by conducting central randomisation stratified for important prognostic factors. Furthermore, we employ blinded assessment of the primary outcome measure and will analyse our data with 'intention to treat'. We are aware of the fact that the secondary outcome measures are at risk of being assessed with some bias favouring the experimental intervention. The risk of random error has been reduced by basing our sample size estimation on conservative estimate regarding the minimal relevant difference between the control and experimental group.

Randomised trials on complex interventions need proper description of the interventions, both before the launch of the trial as well as after the conduct of the trial [52]. In the present article we describe how we intend to apply the interventions in the experimental group and the control group. We will at the six month follow up collect information on how the interventions have been administered in the two intervention groups, making it possible for us to ascribe any significant differences regarding the primary outcome to the interventions provided. Any significant differences regarding the secondary outcomes ought to be interpreted conservatively, first because they will be assessed without blinding and second because they may be due to random errors.

## List of Abbreviations

HE: hand eczema; OHE: occupational hand eczema; ACD: allergic contact dermatitis; ICD: irritant contact dermatitis; AHE: atopic hand eczema; HECSI: Hand Eczema Severity Index; DLQI: Dermatology Life Quality Index; CTU: Copenhagen Trial Unit; BGW: Berufsgenossenschaft fur Gesundheitsdienst und Wohlfahrtspflege; TEWL: transepidermal water loss.

## Competing interests

The authors declare that they have no competing interests.

## Authors' contributions

KI and TA are responsible for the design of the trial. KI is responsible for the protocol and for drafting the manuscript. The coordinating and principle investigator of the trial is KI.

JLH and CG have contributed with important intellectual revision of the design of the trial and protocol, including statistical and ethical aspects. All authors have read and approved the final manuscript.

## Pre-publication history

The pre-publication history for this paper can be accessed here:

http://www.biomedcentral.com/1471-5945/10/8/prepub
